# Pulsed Electric Field-Modified Hot-Pressed Peanut Meal Protein for Gel-like High Internal Phase Emulsions

**DOI:** 10.3390/gels12070571

**Published:** 2026-06-29

**Authors:** Yutong Liao, Jiayi Song, Jiaxin Huang, Kexin Liang, Zichen Song, Zhibo Liang, Ming Yu, Di Zeng, Siming Zhu

**Affiliations:** 1 School of Food Science and Engineering, Guangdong Ocean University, Yangjiang Campus, Yangjiang 529500, China; yutongliao@foxmail.com (Y.L.); jiayisongsing@foxmail.com (J.S.); jasyh@foxmail.com (J.H.); liangkexin03@163.com (K.L.); songzichenszc@foxmail.com (Z.S.); lzhiboy@126.com (Z.L.); yuemin1973@126.com (M.Y.); lfsmzhu@scut.edu.cn (S.Z.); 2 School of Food Science and Engineering, South China University of Technology, Guangzhou 510641, China; 3 Yangjiang Research Institute of Guangdong Ocean University, Guangdong Ocean University, Yangjiang 529500, China

**Keywords:** hot-pressed peanut protein isolate (HPPI), pulsed electric field (PEF), interfacial activity, emulsifying properties, emulsion gel, high internal phase emulsions (HIPEs)

## Abstract

Hot-pressed peanut protein isolate (HPPI), severely denatured during oil extraction, exhibits limited interfacial functionality, restricting its application in structured emulsions. In this study, high-voltage pulsed electric field (PEF) was employed to modulate the structural and interfacial properties of HPPI, a sustainable food biopolymer. PEF treatment induced conformational rearrangement, including a shift in secondary structure from α-helix to β-sheet and increased exposure of hydrophobic residues. These structural changes reduced particle size and increased surface charge, with optimal modification at 2.5 kV/cm. Consequently, interfacial activity was significantly improved, as evidenced by decreased interfacial tension and increased dilatational modulus, indicating a more elastic interfacial film was formed. The modified protein (2.5 kV/cm) effectively stabilized high internal phase emulsions (HIPEs) with typical gel-like viscoelastic features, achieving optimal stability at 2.0 wt% protein concentration, 75% oil phase fraction, and NaCl concentrations below 100 mM. Overall, PEF treatment enhances the interfacial functionality of HPPI by modulating its structure and interfacial film properties, thereby facilitating the fabrication of biopolymer-based food-grade HIPEs for practical food applications.

## 1. Introduction

Hot-pressed peanut meal protein, a major by-product of oil extraction, represents an abundant plant protein resource. However, severe thermal denaturation during hot-pressing leads to irreversible aggregation, resulting in limited interfacial adsorption capacity. This fundamentally restricts its functionality in emulsion-based systems, where interfacial behavior governs stability [1].

Pulsed electric field (PEF) is a promising non-thermal technique used to modulate protein structure [2]. By leveraging the polarization effect generated by instantaneous high-intensity electric fields, PEF can precisely induce the conformational rearrangement of protein molecules [3]. It has been reported that PEF treatment enhances surface hydrophobicity and emulsifying properties in a variety of proteins [4,5,6]. However, most studies have focused on native or mildly denatured proteins, and it remains unclear whether PEF-induced structural modification can effectively restore the interfacial functionality of severely denatured proteins [7].

More importantly, structural modification does not necessarily translate into functional recovery at oil–water interfaces. The stabilization of emulsions relies not only on molecular unfolding but also on rapid protein adsorption at the interface, followed by rearrangement and the formation of a cohesive viscoelastic interfacial film [8]. For highly aggregated proteins, even if partial unfolding occurs, limited mobility and hindered interfacial rearrangement may still restrict the formation of an effective interfacial network. Therefore, understanding how PEF-induced structural rearrangement governs interfacial adsorption behavior and interfacial film formation is critical for elucidating the mechanism of functional recovery in denatured protein systems [9].

High internal phase emulsions (HIPEs) are emulsions with a high internal phase volume fraction and are highly sensitive to the interfacial properties of emulsifiers due to their densely packed droplet structure [10]. This feature makes HIPEs an ideal model system for amplifying differences in interfacial functionality and evaluating the effectiveness of protein-based emulsifiers [11,12]. However, due to the limited interfacial activity and weak film-forming ability of HPPI, it is difficult to construct stable HIPEs without appropriate modification, which further highlights the necessity of regulating its interfacial behavior.

This work systematically examines how PEF intensity influences the structural, physicochemical (particle size, zeta potential, and surface hydrophobicity), and interfacial properties of HPPI, aiming to clarify the mechanisms underlying PEF-induced conformational and interfacial changes. On this basis, the modified HPPI was utilized as an emulsifier to construct HIPEs. The influence of protein concentration, internal phase volume fraction, and ionic strength on emulsion stability and network structure was further evaluated. This work is expected to provide insights into the restoration of heat-denatured peanut protein isolate functionality and to contribute to the theoretical understanding and potential application of plant-based HIPE systems.

## 2. Results and Discussion

### 2.1. Structural Properties

#### 2.1.1. FTIR Spectroscopy Analysis

**Figure 1:**
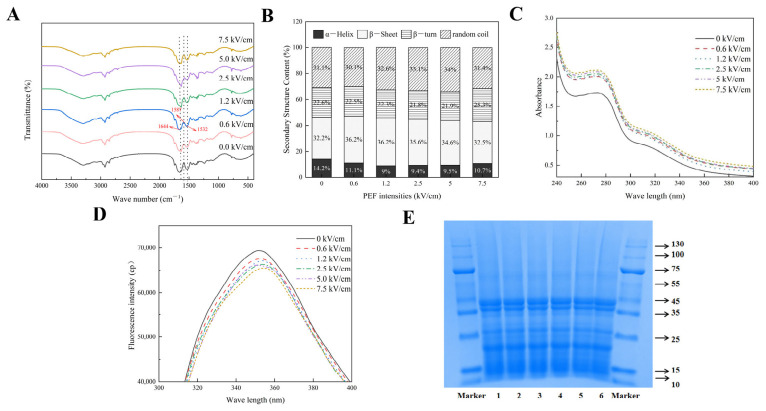
Structural patterns of high-temperature peanut protein isolate (HPPI) treated with different pulsed electric field (PEF) intensities. (**A**) FTIR spectra; (**B**) secondary structure content calculated from FTIR spectra; (**C**) intrinsic fluorescence spectra; (**D**) UV-Vis absorption spectra; (**E**) SDS-PAGE patterns of HPPI. Note: Lanes 1–6: HPPI treated at 0, 0.6, 1.2, 2.5, 5.0 and 7.5 kV/cm, respectively.

FTIR spectroscopy was used to characterize the secondary structure of HPPI under different PEF intensities. As shown in Figure 1A, in the amide I band (1700–1600 cm^−1^, C=O stretching vibration) and amide II band (1600–1500 cm^−1^, N–H bending and C–N stretching vibrations) regions, the absorption bands exhibited a shift toward lower wavenumbers accompanied by band broadening. This indicated that PEF treatment disturbed the hydrogen bonding network maintaining the protein secondary structure, thereby inducing structural rearrangement [13].

Further quantitative analysis of the secondary structures (Figure 1B) revealed that the contents of α-helix and β-turn exhibited a trend of initially decreasing and then increasing with increasing PEF intensity, whereas the β-sheet content showed an opposite trend. This suggested that PEF treatment promoted the interconversion between protein secondary structures, reflecting a transition from the original conformation to a rearranged state rather than a simple disordered process. A similar phenomenon was observed in soybean protein [14], and the mechanism may be associated with the weakening of hydrogen bonds and inducing dipole polarization of functional groups by the electric field, which drives the transition between different secondary structures.

#### 2.1.2. UV-Vis Spectroscopy Analysis

UV-vis absorption spectroscopy was used to monitor changes in protein tertiary structure. As shown in Figure 1C, HPPI exhibited characteristic absorption peaks at approximately 275 nm, corresponding to aromatic amino acid residues, mainly tryptophan (Trp) and tyrosine (Tyr). After PEF treatment, no significant shift in the peak position was observed, indicating that the characteristic absorption band of aromatic residues was largely maintained. However, the absorbance gradually increased with PEF intensity. This suggested that the protein molecules underwent a certain degree of conformational unfolding accompanied by aggregate dissociation, which exposed some aromatic residues previously buried in the molecular interior to the solvent environment [15].

#### 2.1.3. Intrinsic Fluorescence Analysis

Intrinsic fluorescence spectroscopy further revealed the conformational changes in HPPI. As shown in Figure 1D, the position of the maximum emission wavelength remained basically unchanged after PEF treatment, but the fluorescence intensity decreased significantly as the electric field intensity increased, indicating that the tertiary structure of the protein was disturbed. The fluorescence quenching phenomenon suggested that Trp residues became more exposed to a polar microenvironment, thereby strengthening solvent quenching effects [16].

#### 2.1.4. SDS-PAGE Analysis

As presented in Figure 1E, the primary protein bands of HPPI were distributed around 18, 25, 35, and 42 kDa. Compared with the untreated sample, no obvious changes were detected in the position or intensity of the protein bands following PEF treatment, indicating that the molecular weight distribution and subunit composition remained basically stable. Similar results have been observed in other plant protein systems, such as chickpea protein isolate and duck liver globular protein [17,18]. This suggests that PEF treatment primarily influenced the secondary and tertiary structures of the protein, altering its spatial structure by inducing conformational unfolding and rearrangement, but did not disrupt the peptide bond backbone or cause subunit dissociation [19].

### 2.2. Physicochemical Properties and Molecular Interactions

#### 2.2.1. Particle Size Analysis

**Figure 2:**
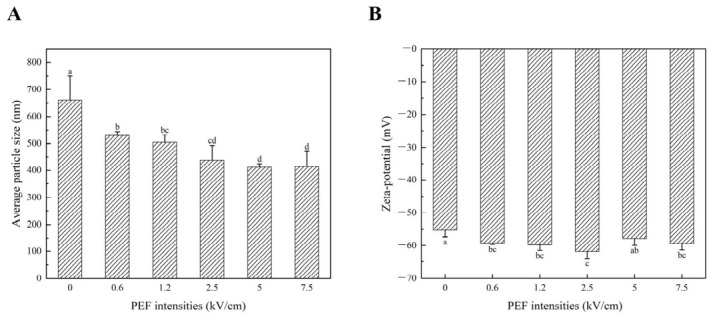
Effect of different PEF treatments on the mean particle size (**A**) and zeta potential (**B**) of HPPI. Significant differences exist between different letters (*p* < 0.05).

Figure 2A presents the changes in particle size of HPPI under different PEF intensities. The untreated sample showed the largest mean particle size. As PEF intensity increased to 5 kV/cm, particle size was significantly reduced (*p* < 0.05) from 660.63 nm to 412.47 nm, indicating that moderate PEF treatment promoted the dissociation of protein aggregates into smaller and more dispersible particles. This reduction may result from the breakdown of non-covalent interactions and conformational loosening of protein molecules, thereby weakening intermolecular associations [20].

However, when the electric field intensity further increased to 7.5 kV/cm, the particle size increased again. This phenomenon may originate from excessive unfolding of protein molecular chains, which strengthened intermolecular non-covalent forces such as hydrophobic attraction and led to re-aggregation, resulting in the formation of larger particles [21].

#### 2.2.2. Zeta Potential Analysis

Zeta potential reflects the surface charge characteristics of particles and the stability of the system. As shown in Figure 2B, all zeta potential measurements were conducted at the native pH of the protein dispersions (approximately pH 7.0). Since this pH was higher than the isoelectric point of peanut protein (pI ≈ 4.5), the protein particles carried net negative charges and therefore exhibited negative zeta potential values [22,23]. After PEF treatment, the absolute value of the zeta potential became more negative as the electric field strength increased, attaining the highest magnitude at 2.5 kV/cm before slightly declining at higher intensities. The enhanced absolute zeta potential indicated an improvement in surface charge density, which enhanced the electrostatic repulsion between particles, promoted dispersion stability, and inhibited the formation of aggregates [24]. At higher electric field intensities, the modest reduction in the absolute zeta potential was likely associated with protein re-aggregation induced by excessive unfolding. The enhanced intermolecular interactions, particularly hydrophobic associations, could lead to the burial or redistribution of charged groups, thereby reducing the effective surface charge and weakening electrostatic repulsion [25].

#### 2.2.3. Surface Hydrophobicity (H_0_)

**Figure 3:**
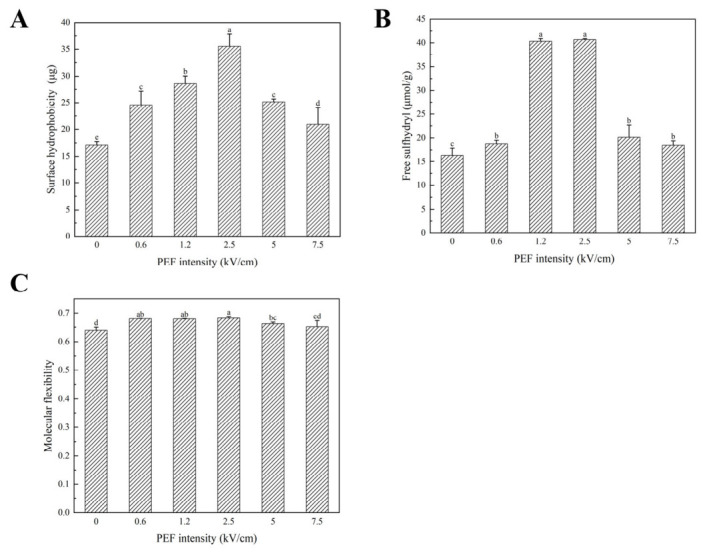
Effects of different PEF intensities on surface hydrophobicity (**A**), free sulfhydryl groups (**B**), and molecular flexibility (**C**) of HPPI. Significant differences exist between different letters (*p* < 0.05).

H_0_ represents the amount of hydrophobic moieties exposed on protein surfaces [26]. As shown in Figure 3A, the H_0_ of HPPI initially increased and subsequently decreased as PEF intensity increased, reaching a maximum at 2.5 kV/cm, where it significantly increased (*p* < 0.05) from 17.13 to 35.56. The increase in H_0_ indicated that PEF induced the conformational relaxation of protein molecules, resulting in the exposure of hydrophobic moieties originally embedded within the protein matrix to the molecular surface, thereby increasing overall hydrophobicity [27]. However, under stronger electric field conditions, excessive treatment might promote the enhancement of hydrophobic interactions, leading to protein re-aggregation. This caused some hydrophobic groups to be re-buried or to make them difficult to bind with the probe, thus resulting in a decrease in H_0_ [28]. Similar trends in other protein systems have also been observed in previous studies [29].

#### 2.2.4. Free Sulfhydryl (SH) Groups

SH groups reflect the conformational changes in proteins and the exposure of internal groups. As shown in Figure 3B, with the increase in PEF intensity, the free SH content of HPPI exhibited an initial increase followed by a decrease, attaining the highest value at 2.5 kV/cm and subsequently decreasing significantly (*p* < 0.05) from 40.67 μmol/g to 18.49 μmol/g.

The increase in free SH content indicated that PEF treatment promoted the unfolding of the protein structure, exposing internal sulfhydryl groups to the molecular surface [30]. At higher electric field intensities, the sulfhydryl groups might undergo oxidation or participate in the formation of disulfide bonds; meanwhile, the strengthened molecular associations and protein aggregation could promote the re-embedding of some sulfhydryl moieties, thereby reducing the free SH level [31].

#### 2.2.5. Analysis of Molecular Flexibility

As shown in Figure 3C, PEF treatment significantly increased the molecular flexibility of HPPI (*p* < 0.05). The increase in molecular flexibility suggested a more dynamic protein conformation, thereby favoring rapid migration and adsorption at the oil–water boundary, as well as effective rearrangement on the interface [32].

Higher flexibility enabled protein molecules to establish a more compact and resilient interfacial layer, thereby reinforcing the cohesiveness and mechanical strength of the interfacial membrane and ultimately enhancing the overall robustness of the emulsion.

The enhanced flexibility suggests that the protein conformation became less rigid, which is indicative of partial unfolding and reduced structural constraints [8]. This conformational change may facilitate molecular mobility and adaptability, enabling proteins to more readily respond to environmental perturbations.

### 2.3. Interfacial Properties and Emulsifying Capacity

#### 2.3.1. Interfacial Tension (IFT)

**Figure 4:**
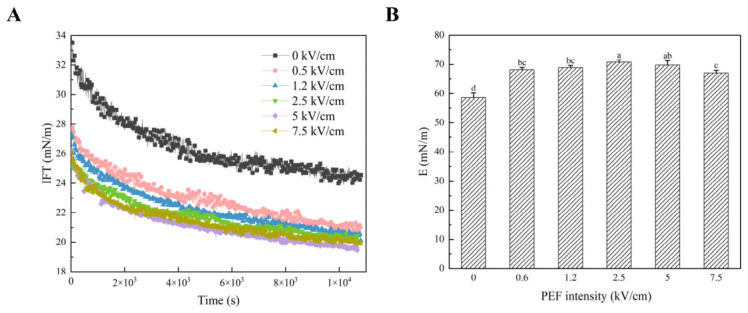
Effects of different PEF intensities on interfacial tension (IFT, (**A**)) and interfacial dilational modulus (E, (**B**)) of HPPI. Significant differences exist between different letters (*p* < 0.05).

IFT reflects the adsorption kinetics and interfacial activity of proteins at the oil–water boundary. As shown in Figure 4A, the interfacial tension of all samples decreased with time and reached adsorption equilibrium after approximately 8000 s. As PEF intensity increased, the equilibrium interfacial tension of HPPI at the interface significantly decreased; however, this decreasing trend leveled off once the electric field strength exceeded 2.5 kV/cm [33].

The reduction in interfacial tension indicates that PEF treatment enhanced the interfacial adsorption capacity of HPPI. This improvement can be correlated with the structural and physicochemical changes observed previously. The decrease in particle size and increased surface charge promoted better dispersion and reduced aggregation, facilitating protein diffusion toward the interface. Meanwhile, the enhanced molecular flexibility suggested a more adaptable conformation, enabling faster adsorption and more effective rearrangement at the oil–water boundary. These combined effects facilitated the development of a more efficient interfacial layer, thereby lowering the interfacial tension [34].

Notably, when the PEF intensity exceeded 2.5 kV/cm, the reduction in interfacial tension became less pronounced, which was likely related to partial re-aggregation and reduced net charge at the particle surface, as evidenced by the particle size and zeta potential results.

#### 2.3.2. Interfacial Viscoelasticity

As shown in Figure 4B, PEF treatment markedly altered the dilatational modulus (E) of the HPPI interfacial film. As the electric field strength increased, the E value gradually increased and reached a maximum at 2.5 kV/cm, suggesting that the protein developed a denser and more resilient interfacial network [35].

This phenomenon suggested that PEF treatment enhanced the interfacial activity of the protein, enabling it to rapidly adsorb and effectively rearrange at the interface, forming a dense and continuous interfacial network structure [36].

Meanwhile, the moderately increased surface charge helped maintain appropriate intermolecular repulsion, preventing the collapse of the interfacial film. Coupled with the improved conformational flexibility, this promoted the dynamic reconstruction of the interfacial layer, thereby reducing film defects and enhancing its resistance to deformation [37]. Nevertheless, when the electric field intensity further increased to 7.5 kV/cm, the E value decreased. This indicated that excessive PEF treatment might lead to over-aggregation or intermolecular association among protein molecules, reducing interfacial film flexibility and making it difficult to effectively respond to interfacial perturbations, thus weakening its viscoelasticity [38].

#### 2.3.3. EAI and ESI

**Figure 5:**
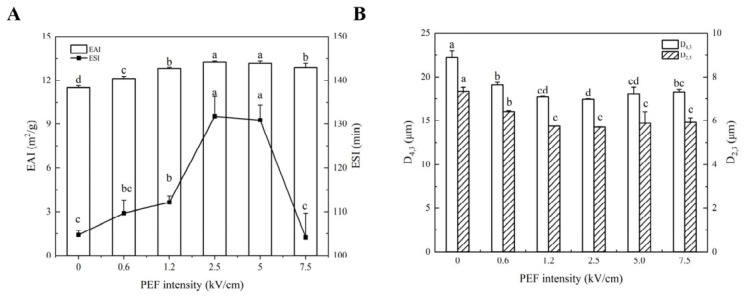
Effects of different pulsed electric field (PEF) intensities on the emulsifying properties and emulsion characteristics of high-temperature peanut protein isolate (HPPI). (**A**) Emulsifying activity index (EAI, left axis) and emulsifying stability index (ESI, right axis); (**B**) Volume-weighted mean diameter (D_4,3_, left axis) and surface-weighted mean diameter (D_2,3_, right axis) of emulsions. Different lowercase letters indicate significant differences between groups (*p* < 0.05).

Figure 5A illustrates the effects of different PEF intensities on the emulsifying properties of HPPI. The EAI reflects the ability of proteins to form emulsions, whereas the ESI reflects the ability of emulsion droplets to remain stable over a long period [19]. The EAI and ESI of HPPI exhibited an initial increase followed by a decrease as electric field strength increased, reaching their maximum values obtained at 2.5 kV/cm. Specifically, the EAI and ESI significantly increased (*p* < 0.05) from 11.50 m^2^/g and 104.69 min to 13.26 m^2^/g and 131.79 min, respectively.

These findings indicated that moderate PEF treatment may significantly enhance emulsifying capacity and emulsion stability of the protein. The primary reason was that the moderate unfolding of the protein conformation increased the exposure of hydrophobic groups, thereby improving its adsorption capacity at the oil–water boundary [39]. Additionally, the reduction in particle size contributed to a more even organization of proteins at the interface, improving the interface coverage efficiency. Meanwhile, the elevated surface charge strengthened electrostatic repulsion among droplets, which effectively inhibited droplet aggregation and improved emulsion stability [40].

When the PEF intensity was excessively high, the protein might undergo over-aggregation or structural rearrangement, leading to a decline in interfacial adsorption capacity, which in turn resulted in a reduction in emulsifying performance.

#### 2.3.4. Emulsion Particle Size

Emulsion droplet size serves as an important parameter for assessing emulsion stability. Figure 5B presents the particle size results of the emulsions. The untreated sample exhibited a larger droplet size, indicating a higher degree of aggregation. As PEF intensity increased, the emulsion particle sizes (D [4,3] and D [2,3]) exhibited an initial decrease followed by an increase, reaching their minimum values at 2.5 kV/cm. Specifically, D [4,3] decreased from 22.22 μm to 17.43 μm, and D [2,3] decreased from 7.34 μm to 5.71 μm (*p* < 0.05).

The reduction in particle size indicated that moderate PEF treatment could promote droplet breakage and improve dispersion uniformity, thereby enhancing emulsion stability [41]. Smaller droplet sizes are beneficial for increasing the specific surface area, enabling proteins to be distributed more efficiently within the interfacial region and generate resilient interfacial layers [42]. However, at higher electric field intensities, the emulsion particle size increased again, which might be due to the decrease in interfacial coverage capacity caused by over-aggregation of proteins, leading to the coalescence and flocculation of droplets and thus reducing emulsion stability.

### 2.4. Formation and Stability of High Internal Phase Emulsions

According to the findings described above, PEF treatment at 2.5 kV/cm markedly enhanced the structural characteristics and interfacial functionality of HPPI, highlighting its promise as an effective stabilizer for high internal phase emulsions (HIPEs). To evaluate whether the enhanced interfacial properties can be translated into macroscopic emulsion performance, HPPI treated at 2.5 kV/cm (PEF-HPPI) was used as an emulsifier to construct HIPEs. The effects of protein concentration, oil phase volume fraction, and ionic strength on the microstructure, rheological properties, and stability of the emulsions were systematically investigated.

#### 2.4.1. Effect of Protein Concentration on HIPEs

HIPEs were prepared at pH 3 with a 75% oil phase using PHPPI at concentrations of 0.5, 1.0, 1.5, 2.0, and 3.0 wt%. All samples remained stationary upon inversion, exhibiting self-standing characteristics and forming viscoelastic semi-solid emulsions [43].

**Figure 6:**
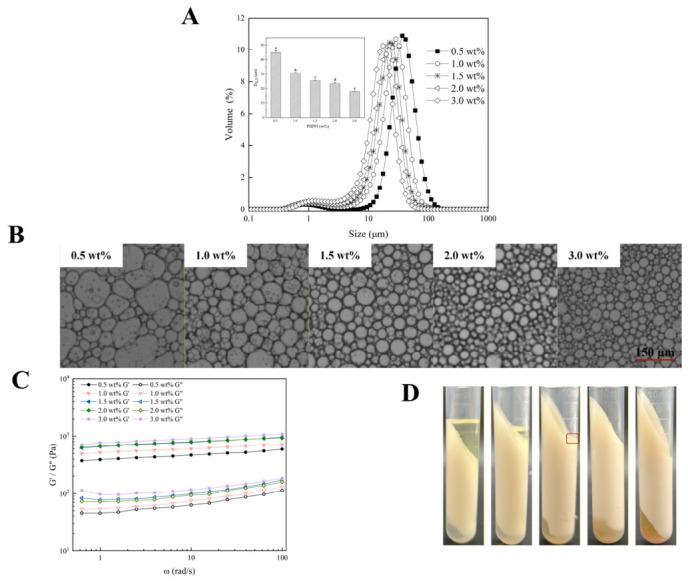
Characterization of high internal phase emulsions (HIPEs) stabilized by different concentrations of high-temperature peanut protein isolate (HPPI). (**A**) Particle size distribution (inset: D [4,3] values); (**B**) Microstructure of HIPEs (scale bar: 150 μm); (**C**) Frequency sweep curves (G′: storage modulus, G″: loss modulus); (**D**) Centrifugal stability of HIPEs, where the red rectangular box marks the oil layer separated from the emulsion after centrifugation. Different lowercase letters indicate significant differences between groups (*p* < 0.05).

Particle size distribution (Figure 6A,B) showed that with increasing protein concentration, droplet size decreased and distribution became more uniform, indicating improved dispersibility. This was likely associated with enhanced protein accumulation at the oil–water boundary, generating a more continuous interfacial network and suppressing droplet coalescence [44].

Consistently, rheological results (Figure 6C) showed that G′ was consistently higher than G″, indicating gel-like behavior. Both G′ and G″ increased markedly (*p* < 0.05) as protein concentration increased, suggesting the development of a denser network structure [45].

Centrifugal stability (Figure 6D) improved with increasing protein concentration, and no oil precipitation was observed at 2.0 wt%, indicating enhanced system stability due to the formation of a stable interfacial film [46].

#### 2.4.2. Effect of Oil Phase Volume Fraction on HIPEs

**Figure 7:**
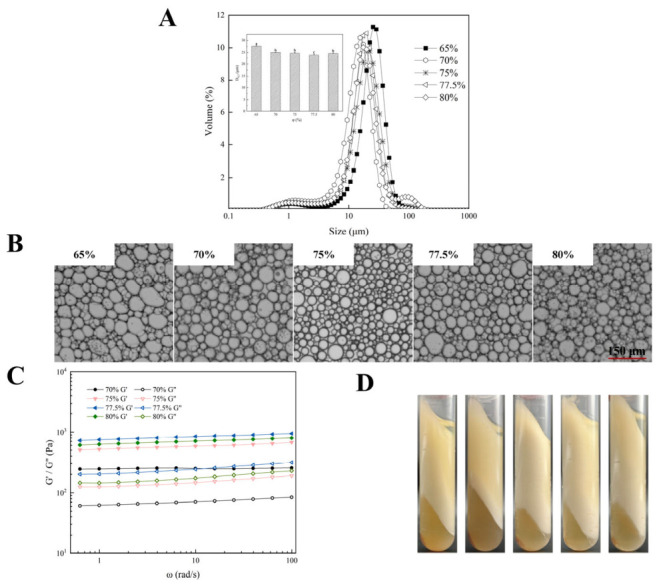
Characterization of high internal phase emulsions (HIPEs) stabilized by HPPI with different oil phase volume fractions. (**A**) Particle size distribution (inset: D [4,3] values); (**B**) Microstructure of HIPEs (scale bar: 150 μm); (**C**) Frequency sweep curves (G′: storage modulus, G″: loss modulus); (**D**) Centrifugal stability of HIPEs. Different lowercase letters indicate significant differences between groups (*p* < 0.05).

The impact of oil phase volume fraction on HIPE characteristics was assessed at pH 3 under a protein concentration of 2 wt%. As shown in Figure 7A,B, with the increase in the oil phase volume fraction, the emulsion particle size gradually decreased and reached a minimum at 77.5%. Optical microscopy observations were consistent with these results, showing that the droplet size decreased, accompanied by a more compact droplet arrangement, indicating the formation of a denser packing structure within the system. However, when the oil phase volume fraction further increased to 80%, the particle size increased instead. This is likely attributable to the excessively high viscosity of the system, which hindered the effective transmission of shear forces; meanwhile, the protein content was relatively insufficient to stabilize all oil droplets, thereby triggering droplet coalescence [47].

Rheological results (Figure 7C) showed that G′ was consistently higher than G″. G′ increased significantly with increasing oil phase volume fraction up to 77.5%, indicating enhanced structural strength of the system [48]. When the oil phase further increased to 80%, both G′ and G″ decreased, suggesting that the internal structure of the system was weakened.

Centrifugal stability (Figure 7D) first increased and then decreased, with optimal stability at 75%. Excessive oil phase led to interfacial film rupture and instability.

#### 2.4.3. Effect of NaCl Concentration on HIPEs

**Figure 8:**
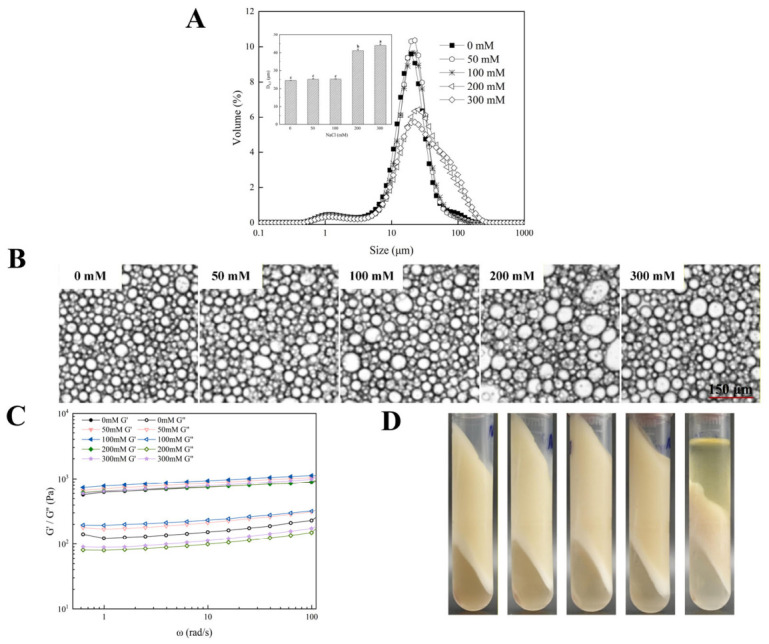
Characterization of high internal phase emulsions (HIPEs) stabilized by HPPI in the presence of different NaCl concentrations. (**A**) Particle size distribution (inset: D [4,3] values); (**B**) Microstructure of HIPEs (scale bar: 150 μm); (**C**) Frequency sweep curves (G′: storage modulus, G″: loss modulus); (**D**) Centrifugal stability of HIPEs. Different lowercase letters indicate significant differences between groups (*p* < 0.05).

The effect of NaCl concentration on the stability of HIPEs is shown in Figure 8. In the range of 0–100 mM, the emulsion particle size showed little change, with a concentrated distribution and uniform droplet size (Figure 8A,B), indicating good stability of the system. With a further increase in NaCl concentration, the particle size distribution progressively broadened, and the proportion of large droplets increased, with D [4,3] significantly increasing from 24.59 μm to 40.29 μm, suggesting the occurrence of emulsion aggregation. This was primarily due to the charge screening effect triggered by elevated ionic strength, which diminished electrostatic repulsion between droplets and thus promoted aggregation [49].

Rheological results (Figure 8C) showed that G′ and G″ exhibited an initial rise and subsequent drop along with the elevation of NaCl concentration, reaching their maximum values at 100 mM. An appropriate amount of salt ions helped regulate intermolecular interactions, promoting the formation of the interfacial film and enhancing the network structure [50]. However, excessively high salt concentrations destroyed the system structure, leading to a decline in viscoelasticity [51].

Centrifugal stability (Figure 8D) remained stable at 0–100 mM, while oil precipitation appeared at 200 mM and severe demulsification occurred at 300 mM, indicating that high ionic strength weakened electrostatic repulsion and destabilized the emulsion.

These results collectively demonstrate that the interfacial functionality of PEF-modified HPPI governs the formation, mechanical strength, and stability of HIPEs through its control over interfacial film formation and droplet network structure.

## 3. Conclusions

This study demonstrates that moderate PEF modification effectively reconstructs interfacial properties of hot-pressed peanut protein by inducing conformational rearrangement and enhancing molecular flexibility. The resulting improvements in interfacial adsorption and film viscoelasticity promote the formation of a more cohesive interfacial layer, which enhances emulsion stability. Under optimal conditions (2.5 kV/cm), the modified protein successfully stabilizes gel-like high internal phase emulsions (HIPEs) by constructing dense droplet networks and robust interfacial films. The overall stability of the system relies on the synergistic balance of interfacial coverage, droplet packing, and electrostatic interactions. These observations suggest new mechanistic understanding of structural–interfacial function interplay between thermally denatured plant proteins and offer a feasible strategy for designing biopolymer-based emulsifiers for food-grade colloidal systems. This work provides theoretical foundations for the functional restoration of thermally damaged peanut protein and its use in developing plant-based structured food gels.

## 4. Materials and Methods

### 4.1. Materials and Reagents

Hot-pressed peanut meal and peanut kernels were obtained from Guangdong Moyang Hua Grain and Oil Co., Ltd. (Yangjiang, China). MCT oil (medium-chain glycerol triacylglycerol: purity > 99.9%) was purchased from Shanghai Pansum Trading Co., Ltd. (Shanghai, China). Corn oil was purchased from Changshouhua Foods Co., Ltd. (Zouping, China). Precast PAGE Gels (Hepes, 4–20%), Prestained Color Protein Marker (10–170 kDa), Coomassie Blue Fast Staining Solution, and Hepes Electrophoresis Buffer were purchased from Beyotime Biotechnology (Shanghai, China). Potassium bromide (HPLC grade), bromophenol blue, glycine, urea, and PC300 (food-grade preservative) were obtained from Shanghai Macklin Biochemical Co., Ltd. (Shanghai, China). All other chemicals used were of analytical grade and obtained from the same supplier unless otherwise specified.

### 4.2. Preparation of HPPI

The hot-pressed peanut meal powder was mixed with deionized water at a ratio of 1:10 (*w*/*v*). The pH of the mixture was adjusted to 9.5 using 1 M NaOH, followed by incubation in a water bath at 60 °C for 2 h. Subsequently, the mixture was centrifuged at 10,000× *g* for 30 min at 4 °C (Avanti J-E, Beckman Coulter, Brea, CA, USA) to collect the aqueous phase. The pH of the supernatant was then adjusted to 4.5 with 1 M HCl, and the mixture was allowed to settle for 30 min. The precipitate was re-dispersed in deionized water, neutralized to pH 7.0 with 1 M NaOH, and dialyzed against deionized water at 4 °C overnight using a cellulose membrane (MWCO 3500 Da; Solarbio Science & Technology Co., Ltd., Beijing, China). Finally, the dialyzed sample was lyophilized using a freeze-dryer (Scientz-10N/A, Ningbo Scientz Biotechnology Co., Ltd., Ningbo, Zhejiang, China) to obtain the HPPI.

### 4.3. PEF Treatment

The HPPI was dissolved in deionized water to obtain a 4% (*w*/*v*) protein solution, followed by stirring at 800 rpm for 2 h at room temperature to achieve uniform hydration. Subsequently, the sample was processed using a pulsed electric field system (PEF-SY-12K, Paihu Technology Co., Ltd., Wuhan, Hubei, China) under the following conditions: pulse voltage of 0–12 kV, pulse frequency of 1–300 Hz, and pulse width of 1–10 μs. The 200 mL protein sample was fed into the continuous PEF system at a flow rate of 60 mL/min via a peristaltic pump. The system was equipped with a continuous four-stage treatment chamber featuring an electrode gap (d) of 6 mm. The electric field strengths employed in this study were 0.6, 1.2, 2.5, 5.0, and 7.5 kV/cm. The pulse width (*P*) and pulse frequency (*f*) were fixed at 7 μs and 60 Hz, respectively. The residence time in the treatment chamber (*t*_V_), the number of pulses (*N*_P_), and effective treatment time (*t*) were calculated according to Equations (1)–(3). The total treatment time was 5 min, corresponding to an effective treatment time of 0.43 ms and a total of 15.2 pulses [25]. The HPPI sample without PEF treatment (0 kV/cm) served as the blank control. All samples were freeze-dried (Scientz-10N/A, Ningbo Xinzhi, China) and stored at −80 °C for subsequent analysis.

(1) $$t_{V}=V/\Phi$$

(2) $$N_{P}=t_{V}\;\times \;f$$

(3) $$t=N_{P}\;\times \;N\;\times \;P$$

where *t*_V_ is the residence time in the chamber, *Φ* is the flow rate (m^3^/s), *V* is the chamber volume (0.17 cm^3^), *N*_P_ is the number of pulses, *f* is the pulse frequency (Hz), *N* is the number of treatment chambers, *P* is the pulse width (s), and *t* is the effective treatment time (s).

### 4.4. Structural Properties Analysis

#### 4.4.1. FTIR Spectroscopy

Prior to analysis, KBr was thoroughly dried. The dried KBr was then mixed with the protein samples at a mass ratio of 100:1 and ground uniformly before being pressed into pellets. FTIR spectra were acquired on an FTIR spectrometer (INVENIO-S, Bruker Optics GmbH, Ettlingen, Germany) at a resolution of 4 cm^−1^ over the wavenumber range of 4000–400 cm^−1^ with 32 scans for both the sample and background. All spectra were processed for baseline correction and smoothing.

#### 4.4.2. Ultraviolet–Visible (UV-Vis) Spectroscopy

The UV-vis absorption spectra were measured using a UV-vis spectrophotometer (Lambda 365+, PerkinElmer, Waltham, MA, USA). The protein solution (1 mg/mL) was transferred into a quartz cuvette with a 1 cm path length. The scanning was performed from 200 to 400 nm at a scanning speed of 800 nm/min [52].

#### 4.4.3. Fluorescence Spectroscopy

Fluorescence spectra of the protein samples (1 mg/mL) were recorded using a fluorescence spectrophotometer (FL8500, PerkinElmer, Waltham, MA, USA). The experimental wavelength was set at 290 nm, and the emission spectra were collected from 300 to 400 nm at a scanning speed of 2400 nm/min. Both the excitation and emission slit widths were maintained at 10 nm. The excitation wavelength of 290 nm was selected to preferentially excite tryptophan (Trp) residues while minimizing the contribution of tyrosine residues. The emission spectra were recorded from 300 to 400 nm to monitor changes in fluorescence intensity and maximum emission wavelength, which were used to evaluate alterations in the local microenvironment of Trp residues and the tertiary structure of the protein.

#### 4.4.4. SDS-PAGE Gel Electrophoresis

The HPPI solution (3 mg/mL) was centrifuged at 9500× *g* for 15 min, and the resulting supernatant was mixed with loading buffer at a ratio of 4:1 (*v*/*v*). The mixture was then heated at 95 °C for 7 min in a water bath. After the addition of electrophoresis buffer, 20 μL of the sample and 3 μL of the protein marker (10–170 kDa) were loaded into each well. Electrophoresis was carried out using a power supply (PowerPac™, Bio-Rad Laboratories, Hercules, CA, USA). The voltage was initially set at 80 V until a sharp protein band front formed, and then increased to 120 V until the bromophenol blue tracking dye reached the bottom of the resolving gel. Gels were stained with Coomassie Brilliant Blue R-250 for 2 h and destained overnight until clear protein bands were observed. Gel images were recorded using a chemiluminescence imaging system (ChemiDoc XRS+, Bio-Rad Laboratories, Hercules, CA, Singapore).

### 4.5. Physicochemical Properties Analysis

#### 4.5.1. Particle Size and Zeta Potential

Particle size and zeta potential of the protein samples (1 mg/mL) were measured using a zetasizer (Zetasizer Nano, Malvern Panalytical Ltd., Malvern, Worcestershire, UK) at 25 °C, with ultrapure water as the dispersive medium. The pH of the protein dispersions used for particle size and zeta potential measurements was approximately 7.0, corresponding to the pH after protein extraction and neutralization.

#### 4.5.2. Protein Surface Hydrophobicity

The protein surface hydrophobicity was evaluated using the bromophenol blue (BPB) binding method as described by Shen et al. [53]. Briefly, 1 mL of protein sample (4 mg/mL) was mixed with 200 μL of BPB solution (1 mg/mL) and incubated at room temperature for 1 h. The mixture was then centrifuged at 13,005× *g* for 20 min. The supernatant was diluted 20-fold, and absorbance was measured at 595 nm using a UV-vis spectrophotometer (Lambda 365+, PerkinElmer, USA). A blank control was prepared by replacing the protein sample with PBS buffer (1 mL) and following the same procedure. The amount of BPB bound (μg) was used to represent the surface hydrophobicity and calculated according to the following equation:

(4) $$\mathrm{BPB}\;\mathrm{bound}\;(\mu g)\;=\;200\;\times \;\left(A_{0}\;-\;A\right)/A_{0}$$

where *A*_0_ is the blank absorbance, *A* represents sample absorbance, and 200 represents the amount (μL) of BPB solution added.

#### 4.5.3. Free Sulfhydryl Group (-SH) Content

The free sulfhydryl group (-SH) content was determined using Ellman’s reagent [54]. Protein (0.02 g) was dissolved in 8 mL of Tris-Gly buffer (0.086 M Tris, 0.09 M glycine, and 4 mM EDTA, pH 8.0), stirred for 20 min until fully dissolved, and centrifuged at 6080× *g* for 15 min. The supernatant (3 mL) was mixed with Ellman’s reagent (50 μL, 4 mg/mL) and incubated in the dark at room temperature for 1 h. Absorbance at 412 nm was recorded using a UV-vis spectrophotometer (Lambda 365+, PerkinElmer, USA), with buffer as a blank. The -SH content was calculated according to the equation below.

(5) $$\text{-}\mathrm{SH}\;(\mu \mathrm{mol}/g)=73.53\;\times \;A_{412}/C$$

where *A*_412_ represents the absorbance at 412 nm, *C* is the protein concentration (mg/mL), and 73.53 is the conversion factor derived from the molar extinction coefficient of DTNB.

#### 4.5.4. Molecular Flexibility

Molecular flexibility of the protein was evaluated using a trypsin digestion method with slight modifications [55]. Trypsin was dissolved in Tris-HCl buffer (0.05 M, pH 8.0) to prepare a 1 mg/mL enzyme solution. The trypsin solution was mixed with HPPI solution (1 mg/mL) at a volume ratio of 1:4 (*v*/*v*) and incubated in a water bath at 38 °C for 10 min. The enzymatic reaction was then quenched by adding 4 mL of trichloroacetic acid (TCA) solution (5 mg/mL). After mixing, the mixture was centrifuged at 6080× *g* for 15 min. The supernatant was collected, and absorbance at 280 nm was measured using a UV-vis spectrophotometer (Lambda 365+, PerkinElmer, USA). The absorbance value (A_280_) was used to indicate protein molecular flexibility.

### 4.6. Interfacial Tension and Dilatational Rheology

Interfacial tension of the 1.0 wt% HPPI solution was measured at room temperature using an interfacial dilatational rheometer (VCA11, Dataphysics Instruments GmbH, Filderstadt, Germany). The protein solution was injected via a syringe into the MCT oil phase in an optical cuvette. The droplet images were recorded for 180 min using a CCD camera to monitor the adsorption process. After equilibrium (3 h), interfacial dilatational rheology was conducted at 0.1 Hz with a strain amplitude of 10%.

### 4.7. Functional Properties Analysis

#### 4.7.1. Emulsifying Activity Index (EAI) and Emulsifying Stability Index (ESI)

Emulsifying properties of HPPI were determined using a turbidimetric method [56]. Briefly, 21 mL of protein solution (15 mg/mL) was placed in a tall beaker. While homogenizing with a high-speed homogenizer (T18, IKA Works GmbH & Co. KG, Staufen, Germany), 7 mL of corn oil was gradually added, followed by homogenization at 15,000 rpm for 2 min. Emulsion samples (100 μL) were collected from 0.5 cm above the bottom of the beaker at 0 and 30 min, respectively. Each aliquot was diluted with 5 mL of 0.1% sodium dodecyl sulfate (SDS) solution. The absorbance of the diluted samples was measured at 500 nm (*A*_0_ and *A*_30_) using a UV-vis spectrophotometer (Lambda 365+, PerkinElmer, USA). The EAI (m^2^/g) and ESI (%) were calculated according to the following equations:

(6) $$\mathrm{EAI}\;=\;2\;\times \;2.303\;\times \;A_{0}\;\times \;N\;\times \;1000/\left(\varphi \;\times \;C\;\right)$$

(7) $$\mathrm{ESI}=30\;\times \;A_{0}/\left(A_{30}\;-\;A_{0}\right)$$

where *A*_0_ and *A*_30_ are the absorbance values measured at 0 and 30 min, respectively; *C* is the protein concentration (mg/mL); *φ* represents the oil phase volume fraction (0.25); *N* is the dilution factor; 2 is the optical path correction factor; 2.303 is the conversion factor from natural logarithm to common logarithm; 1000 is the unit conversion factor; and 30 represents the time interval (min) between absorbance measurements.

#### 4.7.2. Measurement of Droplet Size

Droplet size of the emulsions was measured using a laser diffraction particle size analyzer (LT3600 Plus, Zhuhai Truth Optics Co., Ltd., Zhuhai, Guangdong, China). The optical parameters were set as follows: the refractive index of the oil phase and aqueous phase was 1.460 and 1.330, respectively, with an absorption coefficient of 0.001. Measurements were conducted at a stirring speed of 1500 rpm.

### 4.8. Construction and Characterization of HIPEs

#### 4.8.1. Preparation of HIPEs

PEF-treated hot-pressed peanut meal protein (PHPPI) solutions of varying concentrations were prepared, and the pH was adjusted to 3.0 using 2 M glacial acetic acid. The final protein concentrations were set at 0.5, 1.0, 1.5, 2.0, and 3.0 wt%. To prepare HIPEs with a 75% oil phase volume, 25 mL of the aqueous phase and 75 mL of the oil phase were homogenized at 11,000 rpm for 5 min using a high-speed homogenizer. Specifically, a certain amount of PC300 was added at the 4 min of homogenization as a preservative to inhibit microbial growth during storage, followed by an additional 1 min of stirring.

Furthermore, HIPEs with varying oil phase volume fractions (65%, 70%, 75%, 77.5%, and 80%) were prepared using a 2.0 wt% PHPPI solution at pH 3.0. The total system volume was adjusted to 100 mL by varying the ratio of aqueous to oil phase. To investigate the effect of ionic strength, NaCl was added to the 2.0 wt% PHPPI solution (pH 3.0) at final salt concentrations of 0, 50, 100, 200, and 300 mM.

#### 4.8.2. Optical Microscopy of Emulsions

Immediately after preparation, emulsion samples were collected from 1 cm above the bottom of the container using a glass rod and placed onto a clean glass slide. A coverslip was carefully applied to avoid air bubbles. The emulsion microstructure was then observed using a light microscope (ML31, Guangzhou MSHOT Photoelectric Technology Co., Ltd., Guangzhou, Guangdong, China).

#### 4.8.3. Rheological Properties

Rheological properties of HIPEs were determined using a rotational rheometer (MCR 302, Anton Paar GmbH, Graz, Austria) with a parallel plate geometry (25 mm diameter, 1 mm gap) at 25 °C. The following tests were performed:

(1) Apparent viscosity: The shear rate ranged from 0.1 to 100 s^−1^.
(2) Amplitude sweep: The frequency was 1 Hz, and the strain ranged from 0.1% to 100%.
(3) Frequency sweep: Strain was set at 0.1%, and the angular frequency ranged from 0.1 to 100 rad/s.
(4) Time sweep: The experiment was conducted in three stages. The first and third stages were set at a strain of 0.1% for 250 s, while the second stage was performed at a strain of 80% for 150 s.

#### 4.8.4. Centrifugal Stability

To evaluate the centrifugal stability, 8 g of the HIPEs was transferred into a centrifuge tube and centrifuged at 9500× *g* for 10 min. After centrifugation, the samples were immediately observed for phase separation.

### 4.9. Data Analysis

All experimental data were preliminarily processed using Microsoft Excel 2021, and then Origin 2017 software was used for data processing and image generation. Data were subjected to analysis of variance (ANOVA) using SPSS 27 software. Treatments were considered significantly different at *p* < 0.05.

## Data Availability

All data generated or analyzed during this study are available from the corresponding author upon reasonable request.

## References

[1] Zhang H., Xia Y., Li W., Ma X., Chen L., Wang D., Qu C. (2024). Recent processing of peanut protein in food industry: A molecular structure perspective. Int. J. Food Sci. Technol..

[2] Barba F.J., Parniakov O., Pereira S.A., Wiktor A., Grimi N., Boussetta N., Saraiva J.A., Raso J., Martin-Belloso O., Witrowa-Rajchert D. (2015). Current applications and new opportunities for the use of pulsed electric fields in food science and industry. Food Res. Int..

[3] Niu D., Zeng X.-A., Ren E.-F., Xu F.-Y., Li J., Wang M.-S., Wang R. (2020). Review of the application of pulsed electric fields (PEF) technology for food processing in China. Food Res. Int..

[4] Liu Y.-Y., Zhang Y., Zeng X.-A., El-Mashad H., Pan Z.-L., Wang Q.-J. (2026). Effect of Pulsed Electric Field on Microstructure of Some Amino Acid Group of Soy Protein Isolates. Int. J. Food Eng..

[5] Liao Y., Wang M., Mao F., Mai J., Luo Z., Yan J., Yu M., Zeng D., Zhu S. (2026). Comparative Study on the Structural and Interfacial Properties of Proteins Extracted From Peanut Kernels and Hot-Pressed Peanut Meal. J. Food Sci..

[6] Taha A., Casanova F., Šimonis P., Stankevič V., Gomaa M.A.E., Stirkė A. (2022). Pulsed Electric Field: Fundamentals and Effects on the Structural and Techno-Functional Properties of Dairy and Plant Proteins. Foods.

[7] Baldelli A., Shi J., Singh A., Guo Y., Fathordoobady F., Amiri A., Pratap-Singh A. (2024). Effect of high-pressure on protein structure, refolding, and crystallization. Food Chem. Adv..

[8] Marín-Sánchez J., Berzosa A., Álvarez I., Sánchez-Gimeno C., Raso J. (2024). Pulsed Electric Fields Effects on Proteins: Extraction, Structural Modification, and Enhancing Enzymatic Activity. Bioelectricity.

[9] Xiao W., Geng Y., Gao W., Zeng X.-A., Woo M., Ma J., Han Z. (2026). A comprehensive review of the applications of pulsed electric field: Primary emphasis on extraction, modification, and inactivation. Innov. Food Sci. Emerg. Technol..

[10] Zhang M., Li X., Zhou L., Chen W., Marchioni E. (2023). Protein-Based High Internal Phase Pickering Emulsions: A Review of Their Fabrication, Composition and Future Perspectives in the Food Industry. Foods.

[11] Galvão A.M.M.T., Vélez-Erazo E.M., Karatay G.G.B., Furtado G.d.F., Vidotto D.C., Tavares G.M., Hubinger M.D. (2022). High internal phase emulsions stabilized by the lentil protein isolate (Lens culinaris). Colloids Surf. Physicochem. Eng. Asp..

[12] Huang M., Wang Y., Ahmad M., Ying R., Wang Y., Tan C. (2021). Fabrication of pickering high internal phase emulsions stabilized by pecan protein/xanthan gum for enhanced stability and bioaccessibility of quercetin. Food Chem..

[13] Xu D.-Y., Yu B., Xiao W.-H., Han Z., Wang R., Zeng X.-A. (2026). Pulsed electric field facilitated fibrillation of soy protein isolate: Preparation, characterization, and functional properties. Food Hydrocoll..

[14] Liu Y.Y., Zeng X.A., Deng Z., Yu S.J., Yamasaki S. (2011). Effect of pulsed electric field on the secondary structure and thermal properties of soy protein isolate. Eur. Food Res. Technol..

[15] Huang L., Ding X., Li Y., Ma H. (2019). The aggregation, structures and emulsifying properties of soybean protein isolate induced by ultrasound and acid. Food Chem..

[16] Ni X., Li M., Huang Z., Wei Y., Duan C., Li R., Fang Y., Wang X., Xu M., Yu R. (2025). Study on the regulation of tea polyphenols on the structure and gel properties of myofibrillar protein from Neosalanx taihuensis. Food Chem. X.

[17] Wang Y., Wang Y., Li K., Bai Y., Li B., Xu W. (2020). Effect of high intensity ultrasound on physicochemical, interfacial and gel properties of chickpea protein isolate. LWT.

[18] Xu L., Zheng Y., Zhou C., Pan D., Geng F., Cao J., Xia Q. (2021). Kinetic response of conformational variation of duck liver globular protein to ultrasonic stimulation and its impact on the binding behavior of n-alkenals. LWT.

[19] Teng Y., Wang Y., Xu X., Wang R., Chen B., Wang L., Zhan F., Han Z., Li Y., Zhu X. (2025). Enhancement of chickpea protein functionalities through higher-intensity pulsed electric field: Insights into protein aggregations and structural changes. Food Hydrocoll..

[20] Chen Y., Wang T., Zhang Y., Yang X., Du J., Yu D., Xie F. (2022). Effect of moderate electric fields on the structural and gelation properties of pea protein isolate. Innov. Food Sci. Emerg. Technol..

[21] Wang R., Guo P.-F., Yang J.-P., Huang Y.-Y., Wang L.-H., Li J., Lin S.-Y., Sheng Q.-L., Zeng X.-A., Teng Y.-X. (2025). Exploration of the regulatory mechanism of pulsed electric field on the aggregation behavior of soybean protein isolates. Food Hydrocoll..

[22] Chen X., Xu X., Zhou G. (2016). Potential of high pressure homogenization to solubilize chicken breast myofibrillar proteins in water. Innov. Food Sci. Emerg. Technol..

[23] Liu J., Zhang W., Li P., Jiang Z., Yang R. (2020). Isolation of peanut protein aggregates using aqueous extraction processing combined with membrane separation. Int. J. Food Sci. Technol..

[24] Saricaoglu F.T., Gul O., Besir A., Atalar I. (2018). Effect of high pressure homogenization (HPH) on functional and rheological properties of hazelnut meal proteins obtained from hazelnut oil industry by-products. J. Food Eng..

[25] Hu X., Wang H., Hu Y., Tu Z. (2024). Insight into the effects of pulsed electric field on the structure, aggregation characteristics and functional properties of whey proteins. Food Hydrocoll..

[26] Chandrapala J., Zisu B., Palmer M., Kentish S., Ashokkumar M. (2011). Effects of ultrasound on the thermal and structural characteristics of proteins in reconstituted whey protein concentrate. Ultrason. Sonochem..

[27] Li Y.-Q. (2012). Structure Changes of Soybean Protein Isolates by Pulsed Electric Fields. Phys. Procedia.

[28] Jia N., Zhang F., Liu Q., Wang L., Lin S., Liu D. (2019). The beneficial effects of rutin on myofibrillar protein gel properties and related changes in protein conformation. Food Chem..

[29] Wang Q., Wei R., Hu J., Luan Y., Liu R., Ge Q., Yu H., Wu M. (2022). Moderate pulsed electric field-induced structural unfolding ameliorated the gelling properties of porcine muscle myofibrillar protein. Innov. Food Sci. Emerg. Technol..

[30] Wu L., Zhao W., Yang R., Chen X. (2014). Effects of pulsed electric fields processing on stability of egg white proteins. J. Food Eng..

[31] Li W., Zhou Y., Zhang H., Hu M., Lu P., Qu C. (2024). Study on peanut protein oxidation and metabolomics/proteomics analysis of peanut response under hypoxic/re-aeration storage. Food Chem. X.

[32] Chen Z.-L., Li Y., Wang J.-H., Wang R., Teng Y.-X., Lin J.-W., Zeng X.-A., Woo M.-W., Wang L., Han Z. (2023). Pulsed electric field improves the EGCG binding ability of pea protein isolate unraveled by multi-spectroscopy and computer simulation. Int. J. Biol. Macromol..

[33] Li M., Yu H., Gantumur M.A., Guo L., Lian L., Wang B., Yu C., Jiang Z. (2024). Insight into oil-water interfacial adsorption of protein particles towards regulating Pickering emulsions: A review. Int. J. Biol. Macromol..

[34] Heiden-Hecht T., Müller M., Prevost S., Czakkel O., Zolnierczuk P., Schwärzer K., Förster S., Frielinghaus H., Holderer O. (2026). Phospholipids disrupt the interfacial network of proteins at the oil/water interface. J. Colloid Interface Sci..

[35] Marquez R., Salager J.-L. (2025). Measurement Techniques for Interfacial Rheology of Surfactant, Asphaltene, and Protein-Stabilized Interfaces in Emulsions and Foams. Colloids Interfaces.

[36] D’Alessio G., Maldonado-Valderrama J., Castillo-Santaella T.d., Sabatucci A., Francioso A., Pittia P., Di Mattia C.D. (2025). Pea protein’s interfacial behavior and emulsifying capacity as affected by high-pressure homogenization treatments: An in-depth study with dilatational rheology characterization. Food Hydrocoll..

[37] Jin Y., Liu D., Hu J. (2021). Effect of Surfactant Molecular Structure on Emulsion Stability Investigated by Interfacial Dilatational Rheology. Polymers.

[38] Baldino N., Mileti O., Paleologo M.F.O., Lupi F.R., Gabriele D. (2024). Dilatational and Shear Interfacial Properties of Pea Protein Isolate Systems with Transglutaminase at the Air–Water Interface. Macromol.

[39] Zhang L., Wang L.-J., Jiang W., Qian J.-Y. (2017). Effect of pulsed electric field on functional and structural properties of canola protein by pretreating seeds to elevate oil yield. LWT.

[40] Yang Z., Cheng L. (2024). Impact of Ultrasound Emulsification on the Physicochemical Properties of Emulsions Stabilised by Quinoa Protein Isolates at Different pHs. Food Biophys..

[41] Qi B., Ding J., Wang Z., Li Y., Ma C., Chen F., Sui X., Jiang L. (2017). Deciphering the characteristics of soybean oleosome-associated protein in maintaining the stability of oleosomes as affected by pH. Food Res. Int..

[42] Luo L., Cheng L., Zhang R., Yang Z. (2022). Impact of high-pressure homogenization on physico-chemical, structural, and rheological properties of quinoa protein isolates. Food Struct..

[43] Kan X., Dai Z., Chen D., Zeng X., Fan X. (2023). High internal phase emulsion stabilized by whey protein isolate-gum Arabic Maillard conjugate: Characterization and application in 3D printing. Food Hydrocoll..

[44] Li J., Xu X., Chen Z., Wang T., Lu Z., Hu W., Wang L. (2018). Zein/gum Arabic nanoparticle-stabilized Pickering emulsion with thymol as an antibacterial delivery system. Carbohydr. Polym..

[45] Huang L., Xu C., Gao W., Rojas O.J., Jiao W., Guo S., Li J. (2024). Formulation and stabilization of high internal phase emulsions via mechanical cellulose nanofibrils/ethyl lauroyl arginate complexes. Carbohydr. Polym..

[46] Xiao T., Ma X., Hu H., Xiang F., Zhang X., Zheng Y., Dong H., Adhikari B., Wang Q., Shi A. (2025). Advances in emulsion stability: A review on mechanisms, role of emulsifiers, and applications in food. Food Chem. X.

[47] Ji Y., Han C., Liu E., Li X., Meng X., Liu B. (2022). Pickering emulsions stabilized by pea protein isolate-chitosan nanoparticles: Fabrication, characterization and delivery EPA for digestion in vitro and in vivo. Food Chem..

[48] Wei Z., Huang Q. (2019). Edible Pickering emulsions stabilized by ovotransferrin–gum arabic particles. Food Hydrocoll..

[49] Jiang Y., Zhang C., Yuan J., Wu Y., Li F., Li D., Huang Q. (2019). Effects of pectin polydispersity on zein/pectin composite nanoparticles (ZAPs) as high internal-phase Pickering emulsion stabilizers. Carbohydr. Polym..

[50] Dai H., Li Y., Ma L., Yu Y., Zhu H., Wang H., Liu T., Feng X., Tang M., Hu W. (2020). Fabrication of cross-linked β-lactoglobulin nanoparticles as effective stabilizers for Pickering high internal phase emulsions. Food Hydrocoll..

[51] Wang Y., Zhao J., Zhang S., Zhao X., Liu Y., Jiang J., Xiong Y.L. (2022). Structural and rheological properties of mung bean protein emulsion as a liquid egg substitute: The effect of pH shifting and calcium. Food Hydrocoll..

[52] Zhang J.J., Tu Z.C., Wang H., Hu Y.M., Du P.C., Yang Y.P. (2020). Mechanism of the effect of 2, 2′-azobis (2-amidinopropane) dihydrochloride simulated lipid oxidation on the IgG/IgE binding ability of ovalbumin. Food Chem..

[53] Shen H., Zhao M., Sun W. (2019). Effect of pH on the interaction of porcine myofibrillar proteins with pyrazine compounds. Food Chem..

[54] Li X., Wu Y., Li C., Tong S., Zhang L., Jin J. (2024). Improvement in Noodle Quality and Changes in Microstructure and Disulfide Bond Content through the Addition of Pepper Straw Ash Leachate. Foods.

[55] Jin H., Sun Y., Pan J., Fang Y., Jin Y., Sheng L. (2022). Adsorption kinetics of ovalbumin and lysozyme at the air-water interface and foam properties at neutral pH. Food Hydrocoll..

[56] Tang S., Yu J., Lu L., Fu X., Cai Z. (2019). Interfacial and enhanced emulsifying behavior of phosphorylated ovalbumin. Int. J. Biol. Macromol..

